# 2,3-Tri­methyl­ene-7,8-di­hydro­pyrrolo­[1,2-*a*]thieno[2,3-*d*]pyrimidin-4(6*H*)-one

**DOI:** 10.1107/S1600536813017935

**Published:** 2013-07-10

**Authors:** Khurshed Bozorov, Burkhon Elmuradov, Khusnutdin Shakhidoyatov, Haji Akber Aisa, Bakhodir Tashkhodjaev

**Affiliations:** a S. Yunusov Institute of the Chemistry of Plant Substances, Academy of Sciences of Uzbekistan, Mirzo Ulugbek Str. 77, Tashkent 100170, Uzbekistan; b Xinjiang Technical Institute of Physics and Chemistry, Chinese Academy of Science, Urumqi 830011, People’s Republic of China

## Abstract

The title mol­ecule, C_12_H_12_N_2_OS, is planar, with an r.m.s. deviation of 0.04 Å. In the crystal, the N atom adjacent to the carbonyl group is *sp* ^2^-hybridized. The crystal structure is stabilized by π–π stacking inter­actions observed between thio­phene and pyrimidinone rings of *c*-glide-related mol­ecules [centroid–centroid distance = 3.9554 (13) Å] and by C—H⋯π inter­actions, forming an infinite chain along the *c*-axis direction.

## Related literature

For background information on related compounds, see: Ibrahim *et al.* (1996 ▶); Litvinov (2004 ▶). For the synthesis of the title compound, see: Csukonyi *et al.* (1986 ▶); Elmuradov *et al.* (2011 ▶). For its physiological activity, see: Lilienkampf *et al.* (2007 ▶); Moore *et al.* (2006 ▶). For ^1^H NMR and IR spectroscopy of the title compound, see: Bozorov *et al.* (2013 ▶). For the related structures of the thieno[2,3-*d*] pyrimidin-4-one derivatives, see; Lilienkampf *et al.* (2007 ▶).
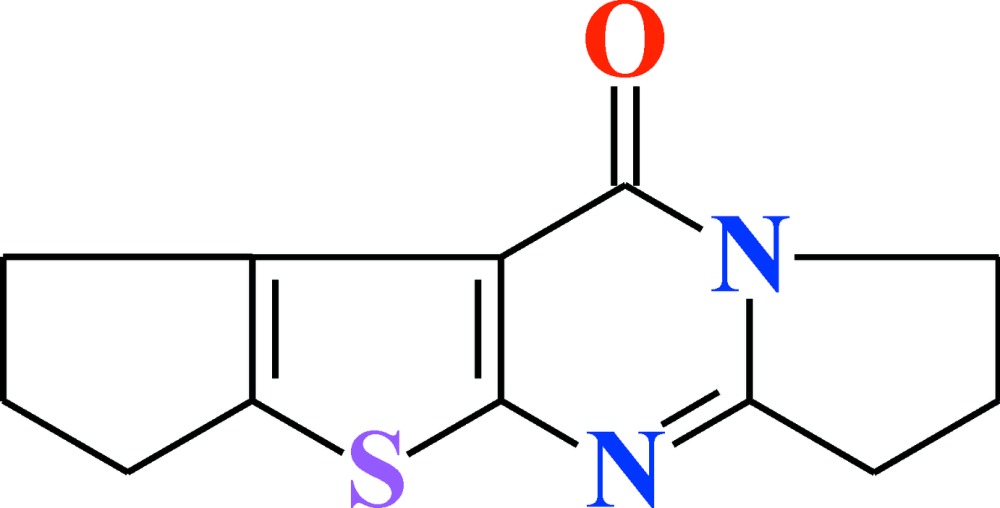


## Experimental

### 

#### Crystal data

C_12_H_12_N_2_OS
*M* *_r_* = 232.30
Monoclinic, 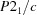

*a* = 10.181 (2) Å
*b* = 12.163 (2) Å
*c* = 8.8624 (18) Å
β = 100.17 (3)°
*V* = 1080.2 (4) Å^3^
*Z* = 4
Cu *K*α radiation
μ = 2.48 mm^−1^
*T* = 292 K
0.20 × 0.17 × 0.15 mm

#### Data collection

Oxford Diffraction Xcalibur Ruby diffractometer
Absorption correction: multi-scan (*CrysAlis PRO*; Oxford Diffraction, 2009 ▶) *T*_min_ = 0.639, *T*_max_ = 0.689
5655 measured reflections
2225 independent reflections
1874 reflections with *I* > 2σ(*I*)
*R*_int_ = 0.022

#### Refinement

*R*[*F* ^2^ > 2σ(*F* ^2^)] = 0.042
*wR*(*F* ^2^) = 0.124
*S* = 1.06
2225 reflections
145 parameters
H-atom parameters constrained
Δρ_max_ = 0.25 e Å^−3^
Δρ_min_ = −0.20 e Å^−3^

### 

Data collection: *CrysAlis PRO* (Oxford Diffraction, 2009 ▶); cell refinement: *CrysAlis PRO*; data reduction: *CrysAlis PRO*; program(s) used to solve structure: *SHELXS97* (Sheldrick, 2008 ▶); program(s) used to refine structure: *SHELXL97* (Sheldrick, 2008 ▶); molecular graphics: *XP* in *SHELXTL* (Sheldrick, 2008 ▶); software used to prepare material for publication: *SHELXTL*, *PLATON* (Spek, 2009 ▶) and *publCIF* (Westrip, 2010 ▶).

## Supplementary Material

Crystal structure: contains datablock(s) I, global. DOI: 10.1107/S1600536813017935/pk2490sup1.cif

Structure factors: contains datablock(s) I. DOI: 10.1107/S1600536813017935/pk2490Isup2.hkl

Supplementary material file. DOI: 10.1107/S1600536813017935/pk2490Isup3.cml

Additional supplementary materials:  crystallographic information; 3D view; checkCIF report

## Figures and Tables

**Table 1:** Hydrogen-bond geometry (Å, °) *Cg*1 and *Cg*2 are the centroids of the S1/C2/C3/C3*A*/C9*A* (thio­phene) and C3*A*/C4/N5/C8*A*/N9/C9*A* rings

*D*—H⋯*A*	*D*—H	H⋯*A*	*D*⋯*A*	*D*—H⋯*A*
C6—H6*B*⋯*Cg*2^i^	0.97	2.85	3.770 (2)	159
C10—H10*B*⋯*Cg*1^ii^	0.97	2.95	3.735 (2)	139

Symmetry codes: (i) 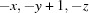; (ii) 
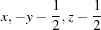
.

**Fig. 1.**
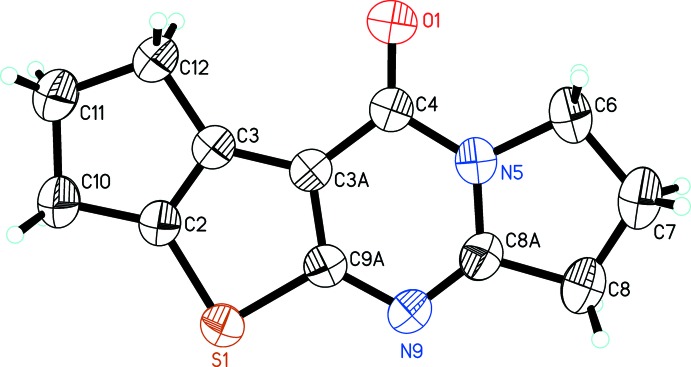
The molecular structure of the title compound. Displacement ellipsoids are drawn at the 50% probability level.

**Fig. 2.**
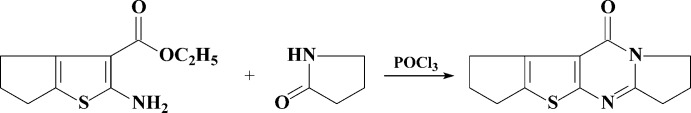
Reaction scheme for the formation of the title compound.

## supplementary crystallographic information

### Comment

Thieno[2,3-*d*]pyrimidin-4-ones (Litvinov, 2004; Ibrahim, *et al.*, 1996) are a large group of heterocyclic compounds. These compounds and their derivatives possess different pharmacological activities (Lilienkampf, *et al.*, 2007; Moore, *et al.*, 2006).

Interaction of ethyl 2-amino-4,5-trimethylene-thiophene-3-carboxylate with γ-butyrolactam in presence of phosphorus oxycloride leads to the formation of a new potentially active tetracyclic thieno[2,3-*d*] pyrimidin-4-one. Synthesis of the title compound was carried out at 368-371 K with reagents in the ratio ester:lactam:POCl_3_ of 1:1.5:3.6. The structure of the synthesized compound has been investigated by XRD analysis.

The molecular structure of the title compound is shown in Fig. 2. As shown in the picture, the molecule of the title compound is planar with r.m.s. deviation of 0.04 Å (non-hydrogen atoms). The sum of bond angles of atom N5 (close to 360°) and bond lengths indicate *sp*^2^ hybridization of the nitrogen atom. This indicates that the lone electron pair of N5 atom participates in a conjugation with π-electrons of carbonyl group (C4═O1).

The crystal structure is stabilized by π-π interactions between neighboring molecules *Cg*1···*Cg*2 [*Cg*1 and *Cg*2 are centroids of the S1/C2/C3/C3A/C9A (thiophene) and C3A/C4/N5/C8A/N9/C9A rings]. With the distance *Cg*1···*Cg2*^i^ of 3.9554 (13) Å [symmetry code: (i) *x*, 1/2 - *y*, 1/2 + *z*] (Spek, 2009). In addition, intermolecular C6–H···*Cg*2 and C10–H···*Cg*1 interactions are present (Table 1).

### Experimental

The title compound was synthesized on the basis of a well-known method (Elmuradov, *et al.*, 2011) (Fig. 2). Light yellow crystals suitable for *X*-ray analysis (in the form of prisms with size 0.20x0.17x0.15 mm) were obtained from DMSO solvent at room temperature, m.p. 475-476 K.

### Refinement

The hydrogen atoms were placed geometrically (with C–H distances of 0.97 Å for CH_2_) and included in the refinement in a riding motion approximation with *U*_iso_=1.2*U*_eq_(C).

### Crystal data

**Table:**

C_12_H_12_N_2_OS	*F*(000) = 488
*M**_r_* = 232.30	*D*_x_ = 1.428 Mg m^−^^3^
Monoclinic, *P*2_1_/*c*	Melting point: 475(1) K
Hall symbol: -P 2ybc	Cu *K*α radiation, λ = 1.54184 Å
*a* = 10.181 (2) Å	Cell parameters from 3341 reflections
*b* = 12.163 (2) Å	θ = 3.6–75.5°
*c* = 8.8624 (18) Å	µ = 2.48 mm^−^^1^
β = 100.17 (3)°	*T* = 292 K
*V* = 1080.2 (4) Å^3^	Prismatic, light yellow
*Z* = 4	0.20 × 0.17 × 0.15 mm

### Data collection

**Table:**

Oxford Diffraction Xcalibur Ruby diffractometer	2225 independent reflections
Radiation source: Enhance (Cu) X-ray Source	1874 reflections with *I* > 2σ(*I*)
Graphite monochromator	*R*_int_ = 0.022
Detector resolution: 10.2576 pixels mm^-1^	θ_max_ = 75.7°, θ_min_ = 4.4°
ω scans	*h* = −12→12
Absorption correction: multi-scan (*CrysAlis PRO*; Oxford Diffraction, 2009)	*k* = −15→14
*T*_min_ = 0.639, *T*_max_ = 0.689	*l* = −9→11
5655 measured reflections	

### Refinement

**Table:**

Refinement on *F*^2^	Primary atom site location: structure-invariant direct methods
Least-squares matrix: full	Secondary atom site location: difference Fourier map
*R*[*F*^2^ > 2σ(*F*^2^)] = 0.042	Hydrogen site location: inferred from neighbouring sites
*wR*(*F*^2^) = 0.124	H-atom parameters constrained
*S* = 1.06	*w* = 1/[σ^2^(*F*_o_^2^) + (0.0783*P*)^2^ + 0.1555*P*] where *P* = (*F*_o_^2^ + 2*F*_c_^2^)/3
2225 reflections	(Δ/σ)_max_ < 0.001
145 parameters	Δρ_max_ = 0.25 e Å^−^^3^
0 restraints	Δρ_min_ = −0.20 e Å^−^^3^

### Special details

**Table:**

Geometry. All e.s.d.'s (except the e.s.d. in the dihedral angle between two l.s. planes) are estimated using the full covariance matrix. The cell e.s.d.'s are taken into account individually in the estimation of e.s.d.'s in distances, angles and torsion angles; correlations between e.s.d.'s in cell parameters are only used when they are defined by crystal symmetry. An approximate (isotropic) treatment of cell e.s.d.'s is used for estimating e.s.d.'s involving l.s. planes.
Refinement. Refinement of *F*^2^ against ALL reflections. The weighted *R*-factor *wR* and goodness of fit *S* are based on *F*^2^, conventional *R*-factors *R* are based on *F*, with *F* set to zero for negative *F*^2^. The threshold expression of *F*^2^ > σ(*F*^2^) is used only for calculating *R*-factors(gt) *etc*. and is not relevant to the choice of reflections for refinement. *R*-factors based on *F*^2^ are statistically about twice as large as those based on *F*, and *R*- factors based on ALL data will be even larger.

### Fractional atomic coordinates and isotropic or equivalent isotropic displacement parameters (Å^2^)

**Table:**

	*x*	*y*	*z*	*U*_iso_*/*U*_eq_	
S1	0.30220 (5)	0.15857 (4)	0.11554 (6)	0.05357 (19)	
O1	0.23418 (14)	0.55482 (10)	−0.02313 (17)	0.0605 (4)	
N5	0.08460 (14)	0.42802 (12)	−0.13884 (16)	0.0443 (3)	
N9	0.09373 (15)	0.23714 (13)	−0.08909 (19)	0.0531 (4)	
C2	0.41506 (17)	0.25495 (15)	0.2035 (2)	0.0463 (4)	
C3	0.38380 (17)	0.35900 (14)	0.15532 (19)	0.0436 (4)	
C3A	0.26442 (16)	0.36454 (13)	0.04359 (18)	0.0417 (4)	
C4	0.19968 (16)	0.45820 (14)	−0.03696 (19)	0.0439 (4)	
C6	0.0044 (2)	0.50792 (17)	−0.2400 (2)	0.0543 (4)	
H6A	0.0578	0.5446	−0.3053	0.065*	
H6B	−0.0338	0.5627	−0.1810	0.065*	
C7	−0.1031 (3)	0.4386 (2)	−0.3334 (3)	0.0831 (8)	
H7A	−0.1903	0.4673	−0.3247	0.100*	
H7B	−0.0942	0.4403	−0.4405	0.100*	
C8	−0.0894 (2)	0.3233 (2)	−0.2749 (3)	0.0686 (6)	
H8A	−0.0839	0.2721	−0.3575	0.082*	
H8B	−0.1647	0.3033	−0.2272	0.082*	
C8A	0.03749 (18)	0.32290 (16)	−0.1596 (2)	0.0485 (4)	
C9A	0.20876 (16)	0.26112 (14)	0.0111 (2)	0.0451 (4)	
C10	0.5408 (2)	0.24589 (17)	0.3183 (2)	0.0588 (5)	
H10A	0.6061	0.2001	0.2806	0.071*	
H10B	0.5240	0.2165	0.4148	0.071*	
C11	0.5869 (2)	0.36625 (19)	0.3350 (3)	0.0713 (6)	
H11A	0.5949	0.3891	0.4411	0.086*	
H11B	0.6737	0.3735	0.3052	0.086*	
C12	0.4854 (2)	0.43942 (16)	0.2330 (2)	0.0571 (5)	
H12A	0.4453	0.4918	0.2938	0.069*	
H12B	0.5268	0.4790	0.1587	0.069*	

### Atomic displacement parameters (Å^2^)

**Table:**

	*U*^11^	*U*^22^	*U*^33^	*U*^12^	*U*^13^	*U*^23^
S1	0.0499 (3)	0.0376 (3)	0.0673 (3)	−0.00227 (17)	−0.0058 (2)	0.00621 (18)
O1	0.0616 (8)	0.0397 (7)	0.0736 (9)	−0.0042 (6)	−0.0058 (7)	0.0034 (6)
N5	0.0413 (7)	0.0445 (8)	0.0446 (7)	0.0029 (6)	0.0010 (6)	0.0028 (6)
N9	0.0455 (8)	0.0439 (8)	0.0636 (9)	−0.0057 (6)	−0.0080 (7)	−0.0022 (7)
C2	0.0415 (8)	0.0443 (9)	0.0501 (9)	−0.0001 (7)	−0.0002 (7)	0.0022 (7)
C3	0.0412 (8)	0.0415 (8)	0.0461 (8)	−0.0018 (6)	0.0019 (7)	−0.0006 (7)
C3A	0.0401 (8)	0.0387 (8)	0.0443 (8)	−0.0013 (6)	0.0021 (6)	−0.0012 (7)
C4	0.0425 (8)	0.0412 (8)	0.0459 (9)	−0.0004 (6)	0.0024 (6)	0.0003 (7)
C6	0.0516 (9)	0.0579 (11)	0.0499 (9)	0.0109 (8)	−0.0002 (7)	0.0097 (8)
C7	0.0787 (16)	0.0799 (18)	0.0743 (15)	−0.0024 (13)	−0.0312 (12)	0.0092 (13)
C8	0.0481 (11)	0.0695 (14)	0.0780 (14)	−0.0021 (9)	−0.0167 (10)	−0.0014 (11)
C8A	0.0404 (8)	0.0516 (10)	0.0501 (9)	−0.0021 (7)	−0.0011 (7)	−0.0034 (7)
C9A	0.0420 (8)	0.0392 (8)	0.0517 (9)	−0.0009 (7)	0.0013 (7)	0.0002 (7)
C10	0.0482 (10)	0.0584 (12)	0.0631 (11)	0.0020 (8)	−0.0086 (8)	0.0058 (9)
C11	0.0600 (12)	0.0613 (13)	0.0799 (14)	−0.0044 (10)	−0.0226 (11)	0.0019 (11)
C12	0.0504 (10)	0.0495 (10)	0.0640 (11)	−0.0065 (8)	−0.0101 (8)	−0.0023 (8)

### Geometric parameters (Å, º)

**Table:**

S1—C2	1.7278 (18)	C6—H6B	0.9700
S1—C9A	1.7339 (18)	C7—C8	1.493 (3)
O1—C4	1.226 (2)	C7—H7A	0.9700
N5—C8A	1.367 (2)	C7—H7B	0.9700
N5—C4	1.396 (2)	C8—C8A	1.499 (2)
N5—C6	1.468 (2)	C8—H8A	0.9700
N9—C8A	1.296 (2)	C8—H8B	0.9700
N9—C9A	1.371 (2)	C10—C11	1.536 (3)
C2—C3	1.355 (2)	C10—H10A	0.9700
C2—C10	1.492 (2)	C10—H10B	0.9700
C3—C3A	1.427 (2)	C11—C12	1.532 (3)
C3—C12	1.500 (2)	C11—H11A	0.9700
C3A—C9A	1.389 (2)	C11—H11B	0.9700
C3A—C4	1.441 (2)	C12—H12A	0.9700
C6—C7	1.508 (3)	C12—H12B	0.9700
C6—H6A	0.9700		
			
C2—S1—C9A	90.63 (9)	C7—C8—H8A	110.8
C8A—N5—C4	124.57 (15)	C8A—C8—H8A	110.8
C8A—N5—C6	113.15 (15)	C7—C8—H8B	110.8
C4—N5—C6	122.23 (16)	C8A—C8—H8B	110.8
C8A—N9—C9A	113.28 (15)	H8A—C8—H8B	108.9
C3—C2—C10	114.15 (16)	N9—C8A—N5	125.00 (16)
C3—C2—S1	113.02 (13)	N9—C8A—C8	125.83 (17)
C10—C2—S1	132.81 (14)	N5—C8A—C8	109.16 (16)
C2—C3—C3A	112.72 (15)	N9—C9A—C3A	126.71 (15)
C2—C3—C12	111.10 (15)	N9—C9A—S1	121.22 (13)
C3A—C3—C12	136.18 (16)	C3A—C9A—S1	112.07 (12)
C9A—C3A—C3	111.55 (14)	C2—C10—C11	101.88 (15)
C9A—C3A—C4	118.64 (15)	C2—C10—H10A	111.4
C3—C3A—C4	129.80 (15)	C11—C10—H10A	111.4
O1—C4—N5	120.62 (16)	C2—C10—H10B	111.4
O1—C4—C3A	127.61 (16)	C11—C10—H10B	111.4
N5—C4—C3A	111.77 (15)	H10A—C10—H10B	109.3
N5—C6—C7	103.66 (17)	C12—C11—C10	109.51 (16)
N5—C6—H6A	111.0	C12—C11—H11A	109.8
C7—C6—H6A	111.0	C10—C11—H11A	109.8
N5—C6—H6B	111.0	C12—C11—H11B	109.8
C7—C6—H6B	111.0	C10—C11—H11B	109.8
H6A—C6—H6B	109.0	H11A—C11—H11B	108.2
C8—C7—C6	108.70 (17)	C3—C12—C11	103.34 (16)
C8—C7—H7A	109.9	C3—C12—H12A	111.1
C6—C7—H7A	109.9	C11—C12—H12A	111.1
C8—C7—H7B	109.9	C3—C12—H12B	111.1
C6—C7—H7B	109.9	C11—C12—H12B	111.1
H7A—C7—H7B	108.3	H12A—C12—H12B	109.1
C7—C8—C8A	104.84 (17)		
			
C9A—S1—C2—C3	−0.38 (15)	C9A—N9—C8A—N5	−0.2 (3)
C9A—S1—C2—C10	−178.7 (2)	C9A—N9—C8A—C8	−179.6 (2)
C10—C2—C3—C3A	178.91 (16)	C4—N5—C8A—N9	−0.8 (3)
S1—C2—C3—C3A	0.3 (2)	C6—N5—C8A—N9	176.90 (19)
C10—C2—C3—C12	−0.5 (2)	C4—N5—C8A—C8	178.62 (17)
S1—C2—C3—C12	−179.19 (14)	C6—N5—C8A—C8	−3.7 (2)
C2—C3—C3A—C9A	0.0 (2)	C7—C8—C8A—N9	−174.1 (2)
C12—C3—C3A—C9A	179.3 (2)	C7—C8—C8A—N5	6.5 (3)
C2—C3—C3A—C4	−178.74 (17)	C8A—N9—C9A—C3A	1.6 (3)
C12—C3—C3A—C4	0.5 (4)	C8A—N9—C9A—S1	−178.89 (14)
C8A—N5—C4—O1	−178.76 (18)	C3—C3A—C9A—N9	179.23 (17)
C6—N5—C4—O1	3.7 (3)	C4—C3A—C9A—N9	−1.8 (3)
C8A—N5—C4—C3A	0.6 (2)	C3—C3A—C9A—S1	−0.3 (2)
C6—N5—C4—C3A	−176.96 (15)	C4—C3A—C9A—S1	178.60 (12)
C9A—C3A—C4—O1	179.91 (18)	C2—S1—C9A—N9	−179.18 (16)
C3—C3A—C4—O1	−1.4 (3)	C2—S1—C9A—C3A	0.40 (14)
C9A—C3A—C4—N5	0.6 (2)	C3—C2—C10—C11	−0.4 (3)
C3—C3A—C4—N5	179.35 (17)	S1—C2—C10—C11	177.87 (17)
C8A—N5—C6—C7	−0.8 (2)	C2—C10—C11—C12	1.2 (3)
C4—N5—C6—C7	177.02 (18)	C2—C3—C12—C11	1.3 (2)
N5—C6—C7—C8	4.9 (3)	C3A—C3—C12—C11	−178.0 (2)
C6—C7—C8—C8A	−6.9 (3)	C10—C11—C12—C3	−1.5 (3)

### Hydrogen-bond geometry (Å, º)

*Cg*1 and *Cg*2 are the centroids of the S1/C2/C3/C3A/C9A (thiophene) and C3A/C4/N5/C8A/N9/C9A rings

**Table:**

*D*—H···*A*	*D*—H	H···*A*	*D*···*A*	*D*—H···*A*
C6—H6*B*···*Cg*2^i^	0.97	2.85	3.770 (2)	159
C10—H10*B*···*Cg*1^ii^	0.97	2.95	3.735 (2)	139

Symmetry codes: (i) −*x*, −*y*+1, −*z*; (ii) *x*, −*y*−1/2, *z*−1/2.
